# Annotations

**Published:** 1896-05-16

**Authors:** 


### ANNOTATIONS.

### Friendly Societies and Revaccination.

THE question has arisen at Gloucester whether the Friendly Societies are liable for sick pay to their members when they are incapacitated for work in consequence of their arms being inflamed from vaccination. It appears that some of the societies were not paying under such circumstances, and that a letter was addressed to the secretary of the Manchester Unity asking for instructions on the point, to which the following answer was returned: "The unfortunate members who are suffering from the effects of vaccination are certainly, on obtaining the usual certificate, entitled to the sick benefits of the Lodge, and I am surprised that anyone should imagine they are not so entitled." This is common-sense and satisfactory. Of course it does not mean that vaccination itself is an excuse for sick pay, but that when, from matters arising from it, such as sore arms, a member is unable to work, then he is to be allowed to throw himself on the Lodge. We also gather from the *Friendly Societies' Recorder* that some of the doctors have refused to certify that a candidate for initiation is in a fit state of health to join a Lodge unless he has been recently revaccinated, and that in this action they are upheld by the Lodges which are thus, in the matter of vaccination, putting themselves in line with the Post Office and other Government services.

### Science in Bonds: Quackery Free.

PERSONS who have a leaning towards quackery in medicine, and who think favourably of "patent medicines" and "secret nostrums," are invited to consider side by side these two sets of facts. The Italian Government has issued some new regulations in regard to the preparation of curative serums. According to these, no person, not even a legally qualified physician or surgeon, is to be permitted to open an institution for the manufacture or sale of such serums, except under the authority of the Minister of the Interior, acting under the control of the Superior Council of Health. Not only so, but before the Superior Council of Health can give its sanction every curative serum proposed to be employed must have been adequately experimented with, on animals if possible, and in any case in some university clinical institute of the kingdom recognised by the Superior Council of Health. This is entirely as it should be; and although it restricts the freedom of every medical man in Italy in his pursuit of scientific methods of improving the healing art, yet it is probable that not one intelligent member of the medical profession throughout the Italian kingdom will be found who will protest against such regulations passed in the interests of public safety and health. But now for the other side of the question. Italy is a paradise of quacks. The most notorious cancer-curing scoundrel of modern times flourishes and grows rich there. What steps does the State take to supervise the preparation of his nostrums, or, for that matter, of any single one of the useless or semi-poisonous panaceas which are sold in every town and village? Not a single step of any kind. The man of science works in bonds, and voluntarily submits to bonds, in order to the greater safety of the public, and the surer advancement of true knowledge. The quack, on the other hand, does exactly as he pleases, prepares what he pleases, in any manner he pleases, and sells it as he pleases, no man or public authority saying him "Nay." The same is true, more or less, in this country. Is there not a rising and intelligent member of Parliament anywhere who can see the absurdity and the danger of this state of things?

### A Moment of Intense Horror.

A CASE recently tried in the Court of Queen's Bench presents several points of interest to the neurologist. A railway signal-man received a message from a box further down the line telling him to stop an express train which was just due. Leaning out of his window and waving a red flag he saw the train approaching, with sparks and dust flying from the track and a carriage apparently off the line. The train in this condition was approaching an arch, in going through which it appeared certain that it must be wrecked; and although, fortunately, it was brought to a stand, and no one was materially hurt, the sudden horror of what he had seen, and the shock produced by the vision of a peril so imminent, so utterly unnerved the signalman that he was unable after that to resume his work. The clinical interest of this case arises from its being an instance of a fact which is well known, but is very apt to be forgotten, viz., that "mere nervous shock," without any suggestion of physical injury, will sometimes produce so deep an impression on the nervous system as to incapacitate a man for his ordinary work for a considerable length of time, and maybe to have a permanent influence on his mental life. A short time ago, in an article in the *British Medical Journal* dealing with the effects of nervous strain, an instance was mentioned in which a station-master witnessing an accident fell down dead on the platform, and another in which a signalman, seeing a collision occur near his box, but from no fault of his own, straightway became insane, and had to be removed to an asylum. Now we often hear of obscure nervous derangements with no clear cause following railway accidents, and of strange nervous symptoms complicating such obvious physical injuries as may occur, and many questions are apt to be raised as to the possibility of such phenomena being due to any known degree of concussion or injury of the nervous centres, and not infrequently much doubt is thrown on the *bonâ fides* of plaintiffs in such cases. While, however, quite admitting the frequency with which fraud is at the bottom of claims against railway companies, it does not do to forget that something else besides mere physical injury may result from a railway accident. If terror, a sudden and intense horror, or, as some would say, a "mere nervous shock," without any physical injury at all, will produce long-lasting changes in the mental and nervous mechanism, it would be strange indeed if such changes were not found in patients who, whatever the nature or extent of their other injuries, have gone through the terrible shock of a serious railway accident. From the moment of the first dancing on the rails, through the terrible time when passengers and portmanteaus are being tossed helplessly about, up to the moment when with a final crunch all becomes still, may not be a long time, but, short as it is, it is a spell of the intensest agony and terror which can be conceived, and it would indeed be passing strange if it did not write deeply on many nervous systems its note of horror

